# Development of On-Line High Performance Liquid Chromatography (HPLC)-Biochemical Detection Methods as Tools in the Identification of Bioactives

**DOI:** 10.3390/ijms13033101

**Published:** 2012-03-07

**Authors:** Christiaan J. Malherbe, Dalene de Beer, Elizabeth Joubert

**Affiliations:** 1 Post-Harvest and Wine Technology Division, Agricultural Research Council, Infruitec-Nietvoorbij, Private Bag X5026, Stellenbosch, 7599, South Africa; E-Mails: DBeerD@arc.agric.za (D.B.); JoubertL@arc.agric.za (E.J.); 2 Department of Food Science, Stellenbosch University, Private Bag X1, Matieland, Stellenbosch, 7602, South Africa

**Keywords:** antioxidant, bioactive phytochemicals, biochemical detection, enzyme inhibition, HPLC, receptor affinity

## Abstract

Biochemical detection (BCD) methods are commonly used to screen plant extracts for specific biological activities in batch assays. Traditionally, bioactives in the most active extracts were identified through time-consuming bio-assay guided fractionation until single active compounds could be isolated. Not only are isolation procedures often tedious, but they could also lead to artifact formation. On-line coupling of BCD assays to high performance liquid chromatography (HPLC) is gaining ground as a high resolution screening technique to overcome problems associated with pre-isolation by measuring the effects of compounds post-column directly after separation. To date, several on-line HPLC-BCD assays, applied to whole plant extracts and mixtures, have been published. In this review the focus will fall on enzyme-based, receptor-based and antioxidant assays.

## 1. Introduction

**Figure 1:**
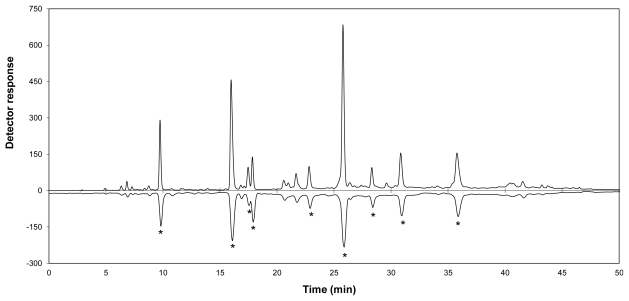
HPLC-diode array detection (DAD) (positive peaks) and on-line 2,2'-azinobis-(3-ethylbenzothiazoline-6-sulfonate) radical cation (ABTS^•+^) scavenging (negative peaks) profiles of an *Athrixia phylicoides* extract (adapted from De Beer *et al.* [4]; * indicates peaks with activity higher than that of caffeic acid).

Historically, plants have provided the basic building blocks for a number of highly effective drugs and they remain an attractive option for discovery of new molecular entities, due to their still largely untapped chemical diversity [1]. About 40% of the chemical scaffolds found in natural products are still absent in today’s medicinal chemistry [2]. The classical approach leading up to a hit or lead compound involves extract preparation and pre-fractionation, biological screening in pharmacologically relevant assays, and isolation and characterization of the active compound(s) through bioassay-guided fractionation [3]. Dereplication techniques allow identification of known compounds responsible for activity of an extract before implementation of bioassay-guided fractionation. Separation of an extract by high-performance liquid chromatography with diode array and mass spectrometric detection (HPLC-DAD-MS) followed by post-column splitting of the HPLC effluent to a fraction collector, allows collection of UV-Vis and MS spectra, as well as fractions that could be screened in an array of *in vitro* assays [1,2]. Recent progress in on-line biochemical detection (BCD), largely as a result of work by groups in the Netherlands (as reviewed in this paper), has seen the development of a number of assays that are used in tandem with HPLC-DAD and/or -MS, classified as high resolution screening methods. BCD assays can be defined as the detection of bioactives based on biochemical reactions or simulated biochemical reactions. These methods are used to fast-track identification of bioactives in extracts or mixtures without the need for lengthy separation and isolation procedures (see Figure 1 for an example). The power of this type of screening procedure is that it cuts down on *in vitro* testing, since only new target compounds, showing activity against the specific disease marker, need to be isolated and tested further. In this review the focus will fall on affinity/activity-based BCD methods, including enzyme activity/affinity detection (EAD), receptor affinity detection (RAD), metabolite profiling systems, and antioxidant activity assays, as tools to identify bioactives in plant extracts amongst others.

## 2. HPLC-BCD Configurations and General Requirements

**Figure 2:**
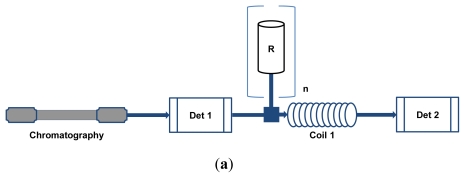

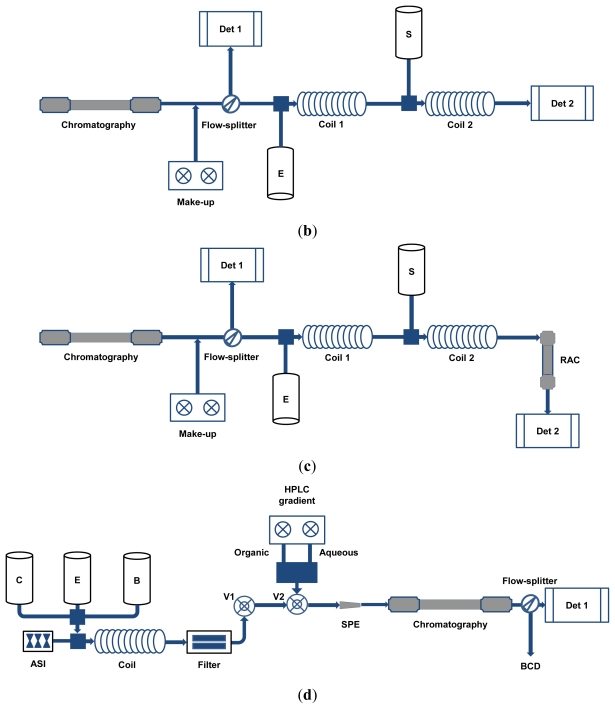
Basic configurations of typical HPLC-BCD systems: (**a**) on-line antioxidant assays; enzyme activity/affinity detection (EAD) and receptor affinity detection (RAD) assays in (**b**) homogeneous configuration and (**c**) heterogeneous configuration; and (**d**) metabolite detection assays [ASI = automatic sample injector; B = buffer; C = cofactor; Det = detector; E = enzyme source/receptor; n = number; R = reagent; RAC = restricted access column; S = substrate; SPE = solid phase extraction; V = valve].

HPLC-BCD instrument configurations vary due to optimization for specific purposes or the preference of the research group involved, but there are several basic configurations that suit particular assay types (Figure 2a–d). The configurations depicted are generalized to fit most assays and variations from these will be discussed in the text. Most of the assays discussed in this review are set up in a post-chromatographic configuration except the assays aimed at identifying bioactive metabolites, which also include pre-chromatographic metabolism of the sample. The simplest configuration, used for most on-line antioxidant assays, has no split in the HPLC effluent and only a single reaction coil (Figure 2a) [5]. Most EAD and RAD assays, as well as some on-line antioxidant assays, make use of flow-splitters to divide the HPLC effluent [6–9]. Part of the flow is directed to the BCD assay, while the rest can be linked to additional detectors used for compound identification or sent to waste. These types of assays can also include pre-incubation of the HPLC effluent with the receptors or enzymes in an incubated coil before the addition of the substrate, followed by a second reaction coil [10]. EAD and RAD assays can further be classified as homogeneous assays (Figure 2b) [11], where there is a marked difference in the signal intensity obtained from bound and unbound ligands, or heterogeneous assays (Figure 2c) [12], where a further separation step is required to distinguish between bound and unbound ligands. The metabolite profiling assays use a configuration where a metabolism system is inserted between the automatic sample injector and the HPLC gradient pump (Figure 2d) [13]. This system, which can be further combined with BCD systems, consists of pumps for addition of the reagents and a reaction coil. After reaction of the sample with the enzyme source and cofactors, the reaction mixture is filtered using a cross-flow filtration device. The reaction mixture is then trapped on a solid-phase extraction (SPE) column prior to HPLC separation. Several valves are also required to allow washing of the filtration device, as well as trapping of the reaction mixture on the SPE column and subsequent elution onto the HPLC column.

Despite the different instrument configurations there are still some general considerations and requirements for the successful development of any of the abovementioned HPLC-BCD assays. Firstly, the chromatographic solvents are rarely compatible with biological material, such as enzymes, receptors and microsomal preparations, and they can interfere in antioxidant reactions. Therefore, make-up flow systems are often employed to either keep the organic modifier content constant through addition of counter-gradients [14] or below noticeable inhibitory concentrations through the addition of buffer as diluent [4]. Secondly, non-specific binding of the biological material to the reaction coil has to be eliminated to increase the resolution in the secondary detector by increasing the signal-to-noise ratio [15]. To this end, an array of additives is available with prior optimization needed to select the most effective additive at the optimal concentration, *i.e.*, detergents, poly-ethylene glycol (PEG) polymers, ELISA blocking agent and bovine serum albumin (BSA) [15]. Use of a coil material that is resistant to binding by enzymes and receptors can also greatly diminish non-specific binding [6,7]. Thirdly, several parameters need to be optimized for sensitivity. These could include reaction times, reaction temperature and suitable buffer systems, depending on the specific reaction. Reaction times may be adjusted via HPLC effluent and reagent flow rate, HPLC effluent split ratio and reaction coil volumes. Furthermore, biological material should be delivered to the HPLC effluent stream in a non-abrasive manner which is best achieved using syringe pumps or Superloops^®^ (Pharmacia) [16]. Peristaltic pumps, despite being prone to fluctuations in pressure, have also been used.

Several detectors can be employed for detection of compounds in the HPLC effluent, the observation of active peaks and the tentative identification of bioactives. In general, any of the substrates, ligands or receptors could be labeled with a chromophore, fluorophore or any other signal delivering probe to enable their detection. In the case of labeled receptors, an affinity column is employed to retain all unbound receptors, hence, if ligands are present, *i.e.*, potential bioactives, this will cause peaks observed on the secondary detector [12]. Many of the basic assay optimization steps are often performed in batch assays, using benchtop detectors for quantification purposes, or flow injection analysis (FIA) mode, *i.e.*, excluding the chromatography step and direct injection of the test solution into the BCD system. In the following sections specific examples illustrating different system requirements will be discussed.

## 3. HPLC Coupled On-Line to EAD and RAD Assays and Their Application in Metabolic Profiling

Hyphenated BCD techniques coupled to HPLC have been developed since the early 1990’s and were broadly divided between EAD and RAD assays. Several reviews have been written over the past decades covering these techniques in general [17–21], or focusing on theoretical foundations of hyphenated assays [22–26] and the implementation of mass spectrometry to strengthen the sensitivity of the assays [27,28]. However, the rather broad description of EAD or RAD does not take into account that some hyphenated techniques are based on the metabolism of compounds and not their affinity for the enzymes responsible for the metabolism. These include systems using cytochromes P450 and liver slices [13,29–31]. Furthermore, there are also assays where HPLC is combined with bio-affinity compounds which may be based on orphan receptors and others based on immunological assays with both types not employing “classical” receptors [32–37]. BCD assays based on enzymes and receptors could thus be divided broadly into four categories: (1) enzyme affinity/activity detection; (2) receptor affinity detection; (3) application of EAD and RAD in metabolite profiling and (4) assays employing antibody interactions. This review will concentrate on the first three categories. The major criteria when evaluating the various assays summarized in this review were sensitivity of the assays to show affinity of compounds for receptors or enzymes and the specificity with which such binding occur. Table 1 summarizes the EAD and RAD assays discussed in this review.

### 3.1. EAD Assays

EAD assays are mainly used in the search for inhibitors of certain enzymes implicated in disease states. There are also some applications where enzyme activities were used to identify proteases and microperoxidases in complex mixtures based on substrate fluorescence. In the current section, assays are grouped in terms of the enzymes under investigation to emphasize the developments that took place within each assay type.

Acetylcholinesterases (AChE) are implicated in diseases such as Alzheimer’s disease, senile dementia, Parkinson’s disease, ataxia and myasthenia gravis [56]. By hydrolyzing acetylcholine into acetate and choline, this enzyme removes the highly active neurotransmitter from the synaptic cleft to prevent overstimulation. However, in the mentioned disease states a diminished secretion of acetylcholine has been implicated as a possible causal factor, thereby focusing research on AChE inhibitors as possible therapeutics.

Ingkaninan *et al.* [6,7] developed a BCD assay based on AChE to search for plant derived AChE inhibitors. Their search was initiated by the hepatotoxic nature of tacrine (tetrahydroaminoacridine), an FDA-approved AChE inhibitor, the discovery of galanthamine from *Narcissus* spp. and its subsequent approval as therapeutic in Austria. Their assay used 5,5'-dithiobis-[2-nitro-benzoic acid] as indicator to react with thiocholine that forms when acetylthiocholine is hydrolyzed by AChE. This reaction causes an increase in absorbance at 405 nm which could be measured by UV-Vis detection. Under assay conditions, buffered in Tris-HCl buffer (50 mM, pH 8) at room temperature, a stable color reaction was achieved with AChE from electric eel (Type VI-S) and inhibitors were observed as negative peaks. All four reagents were added using HPLC pumps in a configuration analogous to Figure 2b, but without the first coil. Neither Tween 80 (polyoxyethylenesorbitan monooleate) nor BSA neutralized non-specific binding, but the use of polyether ether ketone (PEEK), polytetrafluoroethylene (PTFE) and perfluoroalkoxy (PFA) instead of plastic, fluorinated ethylene propylene and Tefzel as coil material showed a marked decrease in background for these assays. All optimization experiments were performed in FIA mode with a knitted PFA coil. Organic modifiers proved to have a marked effect on enzyme activity with 10% acetonitrile or 0.25% acetic acid inhibiting AChE by 90%. The inhibitory effect of 10% methanol, on the other hand, was less than 50%. Even though methanol had an inhibitory effect on AChE, it also decreased non-specific binding in the coil, thereby improving the assay resolution. During this optimization process the known AChE inhibitors, physostigmine and galanthamine, were used and varying concentrations injected to determine the detection limits of the assay. The results were also compared to that obtained with batch assays. When centrifugal partition chromatography (CPC) fractions of methanolic *Narcissus* bulb extracts were analyzed using the HPLC-BCD assay, an extra inhibitory peak was observed [6]. In follow-up work after further purification of ethanolic bulb extracts by CPC and structure elucidation using electrospray ionization (ESI)-MS and ^1^H NMR experiments, the peak was identified as ungiminorine, a mild AChE inhibitor [7]. It was suggested that improvements in the sensitivity might be achieved by BCD based on fluorescence, as well as decreasing band broadening and noise. Fabel *et al.* [38] developed an intricate system, named Segmented Flow Enzyme Inhibition Detection, to decrease band broadening in the secondary detector and improve resolution by segmenting the BCD effluent. To illustrate this system they employed the same colorimetric method devised by Ingkaninan *et al.* [6], but with additional components to enable segmentation of the flow by the addition of air into the BCD effluent.

Rhee *et al.* [39] developed a fluorometric method for the detection of AChE inhibitors in FIA mode. By employing a fluorogenic substrate, 7-acetoxy-1-methyl quinolium iodide (AMQI), which is hydrolyzed to the fluorescent 7-hydroxy-1-methyl quinolium iodide by AChE, they improved the sensitivity of the BCD assay 20-fold. Optimization was performed in FIA mode and showed 70% enzyme activity in the presence of 30% methanol or 5% acetonitrile. Furthermore, a sodium phosphate buffer at pH 7 was used in the assay with the exception of the substrate, which was stored at 0 °C in the same buffer at pH 5 for enhanced stability. The substrate and enzyme were added to the system using peristaltic pumps. BSA was used to eliminate non-specific binding, thereby improving assay resolution. Twenty nine plant extracts of various origins were screened with extracts from bulbs of species from the Amaryllidaceae family showing high inhibitory activity towards AChE. These results were compared with those of batch assays using the colorimetric assay as described by Ingkaninan *et al.* [6]. Marques *et al.* [40] recently used a modification of this fluorogenic method coupled to HPLC to study the AChE inhibitory activity of tacrine metabolites. Their modifications included continuous addition of AChE and AMQI to the HPLC effluent using Superloops^®^ kept on ice. Inhibitory compounds were detected as negative peaks on the chromatogram obtained with fluorescence detection. UV and high resolution quadropole time-of-flight (Q-TOF) MS/MS analyses of the HPLC effluent were used for quantification and identification of compounds, respectively. The study indicated that the synthesis of chemical or biological degradation products of drugs could lead to the improvement of therapeutic treatments. For a more direct method of analysis ESI-MS could be employed to measure the hydrolysis of acetylcholine to acetate and choline by AChE [41]. By continuously monitoring the levels of choline in the BCD effluent by MS a decrease in the activity of the enzyme can be observed, which is then directly correlated to active compounds. This approach was similar to studies where inhibition of cathepsin B, linked to HPLC and high temperature liquid chromatography, was used to illustrate the advantages of MS in BCD [57,58]. Recently, Kool *et al.* [59] devised an assay in the off-line mode for detecting acetylcholine binding protein of fractions spotted into 1536-well plates.

α-Glucosidases are prime targets for anti-diabetic therapeutics in the search for compounds to curb the rise in diabetes mellitus type II [60]. By inhibiting the hydrolysis of polysaccharides to glucose in the intestine, glucose absorption can be limited, thereby lowering the blood glucose content. In recent years a simple spectrophotometric assay based on the reaction of α-glucosidase from the yeast *Saccharomyces cerevisiae* with the chromogenic substrate, *p*-nitrophenyl-α-glucopyranoside (pNPG), has been applied in the search for α-glucosidase inhibitors in plant extracts [61]. In this assay the *p*-nitrophenyl group is spliced from pNPG with a subsequent increase in absorbance at 405 nm. If an extract contains inhibitors, it will cause a decreased absorbance at 405 nm relative to the control containing no inhibitors.

Li *et al.* [14] developed an HPLC-BCD method based on this colorimetric assay using α-glucosidase from yeast and an instrument configuration similar to that depicted in Figure 2b, but simplified by excluding the first coil. Using reversed phase HPLC and DAD for both the HPLC effluent and the BCD, methanolic extracts of pu-erh tea, eagle tea and radix glycyrrhizae were tested for the presence of α-glucosidase inhibitors. ESI-MS detection was used for the tentative identification of observed α-glucosidase inhibitors. A counter-gradient was employed as make-up flow to keep the concentration of the organic modifier (methanol) constant, while the enzyme and substrate were added in a 1:1 ratio to a split flow from the HPLC effluent. Methanol concentrations below 30% did not have a significant effect on α-glucosidase activity, but 30% acetonitrile severely inhibited activity. No additives were used to increase resolution, but a PEEK reaction coil, controlled at 37 °C and giving *ca.* 2 min reaction time, was employed. Two known compounds, (−)-epigallocatechin gallate (EGCG) and (−)-epicatechin gallate, present in pu-erh tea extract, were shown to have potential as α-glucosidase inhibitors. This method was also regarded as a potential quality control strategy for traditional Chinese medicine [42].

Angiotensin I converting enzyme (ACE), a dipeptidylcarboxypeptidase, forms part of an enzymatic system essential to the modulation of blood pressure and is therefore implicated in related diseases, e.g., hypertension, chronic cardiac failure, cardiac infarctions and diabetic nephropathy [62]. In this enzymatic system, renin converts angiogenin to angiotensin I which is then converted by ACE to angiotensin II, a major vasoconstrictor. At the same time ACE also inactivates bradykinin, a vasodilator, which then leads to an increase in blood pressure [62]. ACE inhibitors thus have potential for the alleviation of these disease conditions. Van Elswijk *et al.* [10] developed a fluorescence method for the detection of ACE inhibitors in milk hydrolysates. The HPLC effluent was split towards an ESI Q-TOF MS for quantification and tentative identification, the BCD system for detecting bioactives and a waste line. A fluorogenic substrate, *o*-aminobenzoic acid-phenylalanine-arginine-lysine-dinitrophenol-proline, was employed as it is cleaved by ACE to cause an increase in fluorescence and therefore an inhibitor would cause a decrease in fluorescence [63]. Tween 20 (polyoxyethylenesorbitan monolaurate) as additive against non-specific binding and Tris buffer (pH 7.5) were employed. For increased sensitivity the ACE-containing mixture was allowed to interact with the HPLC effluent for 1 min in the first reaction coil, prior to the addition of the substrate. Thereafter, a second reaction coil was introduced allowing an additional reaction time of 2 min (Figure 2b). A fluorescence detector was used to monitor the biochemical reaction at excitation and emission wavelengths of 320 nm and 420 nm, respectively. PTFE knitted reaction coils were incubated at 50 °C in an HPLC column oven while enzyme and substrate solutions, delivered by Superloops^®^, were maintained at 4 °C. The presence of 20 bioactive peptides with ACE inhibitory potential was observed in the milk hydrolysates tested. Identification of the peptides was based on their Q-TOF MS fragmentation profiles and comparison with a protein database.

Rudzki *et al.* [64] reviewed the use of techniques hyphenating HPLC and gas chromatography with MS to search for ACE inhibitors based on chemical structure. By combining information from a myriad of publications they emphasized the importance of stereochemical purity of synthesized inhibitors since (*S*)-stereo-isomers were active while (*R*)-stereo-isomers were not. The review was aimed at synthesized compounds and their metabolites in biological samples, but it did not include naturally occurring compounds or a biological detection step.

Liver cytochrome P450s (CYPs) are involved in xenobiotic metabolism and are membrane bound enzymes that require nicotinamide adenine dinucleotide phosphate (NADPH) as electron donor for catalytic activity [65]. Making use of this requirement, Kool *et al.* [15] developed a classical EAD assay with β-naphtoflavone (β-NF) induced rat liver microsomes as source of cytochrome P450 1A1/1A2 to investigate ligands without discriminating between inhibitors and activators. Conversion of the substrate, epoxyresorufin, by CYP 1A1/1A2 to resorufin, a fluorescent compound, in the presence of NADPH, allows monitoring of the reaction by on-line fluorescence detection. Further optimization was performed in FIA mode with seven known ligands and provided concentration values needed to obtain 50% inhibition (IC_50_) comparable to the batch assay. Among the detergents (saponin, sodium cholate and Tween 20 and 80) and PEG polymers (PEG 6000 and 3325) tested, most improved the resolution of the assay, but only Tween 20 and 80 did not inhibit the activity of the microsomes. Superloops® were used to deliver the substrate with Tween 20 and β-NF induced microsomes, maintained in potassium phosphate buffer (pH 7.4) containing sodium chloride. A knitted PTFE reaction coil with volume of 25 μL was incubated at 37 °C in an instrument configuration similar to the schematic depiction in Figure 2b, but excluding the first coil. Methanol or acetonitrile could be used as organic solvents for HPLC separation with optimal concentrations of 3–6% and 1–3%, respectively. A make-up counter-gradient was added to the HPLC effluent to maintain organic solvent concentrations at the optimal level. The throughput of this assay was improved by splitting the HPLC effluent to parallel assays for CYP 1A (β-NF induced microsomes), CYP 2B (phenobarbital induced microsomes) and CYP 3A (dexamethasone induced microsomes) [43]. Known inhibitors were used for validation. Tween 20 was used as additive for the CYP 1A assay, but PEG 6000 was required for both the CYP 2B and 3A assays due to tolerance issues. The substrates, epoxyresorufin (CYP 1A), pentoxyresorufin (CYP 2B) and 7-benzyloxy-4-trifluoromethylcoumarin (CYP 3A), were monitored using separate fluorescence detectors, while the analytes separated by HPLC were quantified by UV detection. This was a major step in the direction of higher throughput in combination with high resolution screening methods.

A further development was the replacement of microsomes with recombinant supersomes expressing only human CYP 1A2 [44]. Inhibitors for CYP 1A2 could be important as cancer prevention treatments, since this enzyme has been implicated as a bio-activator of carcinogens in heat processed meat and the conversion of herb alkenylbenzenes to carcinogenic 1'-hydroxymetabolites. Methoxyresorufin was employed as fluorogenic substrate and split flow was maintained with a mass rate attenuator which mixed small volumes of the HPLC effluent with the make-up flow comprising phosphate buffer. Optimization was first performed in batch assays to obtain the optimal methanol and NADPH concentrations. A dual syringe pump delivered the supersomes and NADPH/substrate mixtures separately into a PEEK reaction coil maintained at 37 °C, giving a reaction time of 3.3 min. IC_50_ values for known inhibitors and substrates were determined using FIA mode. Basil and kava kava extracts, known to contain alkenylbenzenes, were shown to contain inhibitors of CYP 1A2, indicating that potentially carcinogenic alkenylbenzene-metabolism could be reduced in herbal mixtures. Recently, Reinen *et al.* [45] used a fluorescence-based EAD assay with allyloxyresorufin as substrate to screen CYP BM3-mutant libraries for differences in affinity. This assay was very sensitive in terms of the ligands tested against the libraries and allowed structure-function analysis based on affinity. A recent general review on developments in search of plant bioactives by Van Beek *et al.* [66] also covered some of the new developments in on-line CYP EAD research.

Glutathione-*S*-transferases (GST) form part of liver metabolism by conjugating glutathione (GSH) with xenobiotics, anticancer drugs, endogeneous lipid peroxidation products, prostaglandins and electrophilic reactive intermediates [67]. GST could therefore be detrimental to cancer therapies by neutralizing anticancer drugs before they reach their targets. General GST inhibitors could be effective against cancer cells, but might also be lethal to normal cells which would then have diminished GST activity. However, GST P1 is known to be overexpressed in tumor cells and therefore could be a primary target for selective inhibitors without affecting metabolism in adjacent cells. A system similar to the parallel assay developed for CYP enzymes has been published for GST from rat liver cytosol (cGST) and recombinant human GST P1 expressed in *Escherichia coli* [16,43]. Substrates for both enzymes were monochlorobimane (MCB) and GSH which form GSH-bimane, a combined product with a strong fluorescent signal. Optimization, performed in batch assays, resulted in the selection of methanol as organic modifier for HPLC separation, PEG 6000 as additive to GSH and MCB, Tween 20 as additive to the counter-gradient and 100 mM phosphate buffer (pH 6.5) as working buffer for the enzyme and substrate solutions. Reaction volumes for GST P1 and cGST were 500 μL and 75 μL, respectively. Superloops^®^ were used as delivery systems for enzymes and substrates to the HPLC effluent, knitted coils were incubated at 37 °C and the reaction was monitored by fluorescence detection. IC_50_ values of known inhibitors and some synthesized derivatives as determined using FIA mode correlated well with results from the batch assay. The experimental set-up allowed discrimination between general inhibitors, affecting cGST, and specific GST P1-inhibitors. Schebb *et al.* [46] modified the cGST system to investigate GSH adducts from the mycotoxin, patulin, for possible GST inhibition. Modifications included two PEEK reaction coils to allow interaction between enzyme and HPLC effluent, and the reaction after addition of the substrate (Figure 2b). The reaction took place at 40 °C, while the HPLC effluent was monitored by UV and ESI-MS detection for quantification and tentative identification, respectively. Of the 15 patulin-GSH adducts, the dihydro-parinone adduct with one GSH moiety and a ketohexanoic acid with two GSH moieties showed marked inhibition.

Proteases represent a large group of mammalian regulatory proteins with serine proteases being important targets for antithrombotic drugs [68]. Serine protease inhibitors were investigated by Schebb *et al.* [47] using an intricate gradient-parking system, employed to maintain a constant concentration of the organic modifier, methanol, without the need for added make-up pumps. This system required the addition of a switching valve and two extra columns, one analytical and one preparative. It allows for the storage of a symmetrical gradient on the preparative column, which is then added as the make-up flow by switching the valve between analyses and reversing flow through the preparative column. Several gradients were tested as a characterization procedure for the parking system. Candidate serine proteases, thrombin and trypsin, were used to digest the substrate, *H*-d-cyclohexylalanine-Ala-Arg-7-amino-4-methylcoumarin, to release 7-amino-4-methylcoumarin, a fluorescent compound. Inhibitors would cause a decrease in fluorescence relative to the control. Superloops^®^ delivered the enzymes and substrates to the HPLC effluent. Enzymes and substrates were prepared in potassium phosphate buffer (pH 7.4) containing both PEG 6000 and ELISA blocking agent as additives. Two tandem PEEK reaction coils were used to first allow interaction between the HPLC effluent and enzymes prior to the addition of the substrate as shown in Figure 2b. IC_50_ values of known inhibitors, determined using FIA mode, were comparable with the results of batch assays. The system was finally validated by screening a small library of serine protease inhibitors.

The same group investigated a system for the identification of proteases in mixtures of snake venom, from *Bothrops moojeni*, and an eye-infective amoeba, *Acanthamoeba castellanii*, which could have some importance in blood coagulation cascades [69]. Unlike the previously described EAD systems, this system focused on identification of active enzymes by adding a mixture of eight substrates, *p-*nitroaniline labeled peptides, and monitoring the absorbance at 405 nm. However, it should be noted that the active enzymes can then be used in conventional EAD systems in search of bioactive compounds, *i.e.*, inhibitors or activators of proteases. The chromatographic step was also set up in buffer systems based on ion exchange or size exclusion chromatography to prevent inactivation or denaturation of possible proteases. No make-up flow and only one Superloop^®^, delivering the substrate mixture to a knitted fluorinated ethylene propylene reaction coil, were required. Trypsin and chymotrypsin were used as candidate proteases to set up and optimize the system, after which snake venom and supernatant from the amoeba homogenate were tested. Chromatographic separation of the BCD effluent was used in conjunction with ESI-MS/MS to measure the levels of the eight substrates in the mixture after the reaction coil and to investigate the selectivity of candidate proteases and fractions. The active fractions from both samples were identified and could be collected for purification. A similar assay, aimed at the detection of human immunodeficiency virus (HIV)-protease inhibitors, was developed by coupling size exclusion chromatography with a BCD system [48,49]. Subtilisin was used as example protease. In this case fluorescence resonance energy transfer was employed as a detection method by using HIV-protease substrate 1, a peptide covalently bound to two chromophores, namely 5-[(2-aminoethyl)-amino]naphthalene-1-sulfonic acid and 4-(4-dimethylaminophenylazo) benzoic acid. The assay was tested with a mixture of inhibitors, aprotinin and 4-(2-aminoethyl)-benzenesulfonyl fluoride hydrochloride, and with non-inhibitory compounds. IC_50_ values determined using FIA mode and on-line BCD were compared to those of batch assays. The assay could be performed in homogeneous mode (Figure 2b) due to the marked difference in fluorescence between substrate and product.

When cytochrome c is subjected to proteolytic digestion, microperoxidases are formed. Microperoxidases retain the heme group of cytochrome c, but can have peptide chains of varying lengths, e.g., MP11 is a microperoxidase with a chain of 11 amino acids linked to the heme group. Microperoxidases are enzymes used in analytical chemistry to catalyze the oxidation of organic substrates by hydrogen peroxide [70]. Haselberg *et al.* [71] developed a dual substrate system for the detection of microperoxidases in proteolytic digests of cytochrome c. 4-(*N*-methylhydrazino)-7-nitro- 2,1,3-benzooxadiazole is converted by microperoxidases to 4-(*N*-methylamino)-7-nitro-2,1,3-benzooxadiazole, which fluoresces in the presence of hydrogen peroxide. Coupling this BCD system to reversed phase HPLC enabled identification of fractions with microperoxidase activity. MP11 was used as a model microperoxidase to optimize the system in terms of pH, acetonitrile concentration and buffer. The active fractions were identified by ESI-MS, using a reference library. MP6 was identified from the digest of bovine heart cytochrome c by proteases from *Streptomyces griseus*. Even though MP6 is a known microperoxidase, its very high activity indicated potential future applications in biosensors and chemiluminescence systems. It should be noted that these microperoxidases could also be used to find possible bioactive compounds by following the same principles as discussed for conventional EAD systems in this review.

Phosphodiesterases (PDE) are implicated in hypertension, vascular conditions and asthma. The enzyme is responsible for hydrolysis of cyclic adenosine and guanosine 3',5' monophosphates (cAMP, cGMP) to AMP and GMP, respectively [72]. Due to a variety of isozymes which differ in substrate specificity, intracellular location, inhibitor specificity and regulation, it would be advantageous to find isozyme-specific inhibitors. Schenk *et al.* [50] developed a PDE-based on-line BCD assay with mant-cGMP as fluorescent substrate and 3-isobutyl-1-methyl-xanthine as known inhibitor during the validation phases using FIA mode. An evaporative light scattering detector was employed to quantify unknown inhibitory compounds present in the HPLC effluent, while DAD and MS were used to quantify known compounds for IC_50_ determinations, as well as for dereplication based on UV and MS spectra. The assay was monitored by fluorescence detection with peaks indicative of inhibitory activity. BSA at a concentration of 0.05% (w/v) was the most effective additive to increase the resolution of the assay. Final testing, performed on plant extracts spiked with known inhibitors, showed that low levels of inhibitor could be detected. Kool *et al.* [73] developed a flow through assay to measure the modulation of cAMP levels by G-protein coupled receptors based on fluorescence polarization (FP). The assay was coupled to the cAMP binding domain of protein kinase A and fluorescein-labeled cAMP and tested against other nucleotides for sensitivity. This assay was proposed as an alternative to radioactive testing methods.

A general on-line BCD assay for the detection of both phosphate consuming and releasing enzymes based on a fluorophore, phosphate binding protein (PBP), was developed by Schenk *et al.* [51]. Kinases and phosphatases are important drug targets involved in the transfer of phosphate. In FIA mode it could be used to measure the activity of phosphate releasing enzymes, but in chromatography mode with the enzyme added to the HPLC effluent, the assay could be effective in finding inhibitors for phosphate binding and releasing enzymes. Validation was performed with alkaline phosphatase as an example of phosphate releasing enzymes and tetramizole, mixed with plant extract, as inhibitor. Hyphenation with TOF-MS provided information for identification of inhibitors, whereas fluorescence detection was used to detect inhibitors, observed as negative peaks. A powerful advantage of this particular assay is that the PBP could distinguish between free phosphates released by enzymes and bound phosphates forming part of many physiological compounds.

Falck *et al.* [52] developed a BCD assay to investigate small molecule inhibitors of p38α mitogen-activated protein (MAP) kinase. Inhibitors of MAP-kinases are primary drug targets as they are implicated in inflammatory response cascades [74]. The on-line p38α MAP-kinase affinity assay was used in parallel with a high resolution ESI ion trap (IT) TOF-MS system to enable very accurate identification of small binding molecules. This system was similar to the system used by de Vlieger *et al.* [8] for their investigation into ligands for both estrogenic receptors α and β, as discussed in more detail in Section 3.2. Furthermore, Falck *et al.* [52] classified MAP kinase inhibitors into 4 groups according to binding specificity: (1) type I binding only to the adenosine triphosphate (ATP)-binding site; (2) type II binding to both the ATP-binding site and an allosteric binding site in close proximity; (3) type III binding only to the allosteric site and (4) type IV binding to neither of these sites. The fluorescent tracer-ligand, SK&F86002 (SKF), used for detection, was a type I inhibitor which showed a marked increase in fluorescence upon binding to MAP-kinases. This allowed for a homogeneous configuration in this HPLC-EAD assay. Competing ligands would cause a decrease in fluorescence which would be observed as negative peaks in the secondary detector. The system was optimized for reaction temperature (25 °C), tracer concentration at saturation, formic acid content (0.01%) in the HPLC effluent and the reaction coil material, *i.e.*, polar covalently modified (PEG-coated) fused silica tubing. Rigorous testing procedures showed this system to be very reproducible which enabled its application in a semi-quantitative manner. Even though IC_50_ values for known inhibitors, calculated using this system, was considerably lower than those calculated in batch assays, the ranking of these inhibitors still followed a similar trend as observed with the batch assays. Finally, their system proved sensitive enough to detect inhibitors of not only type I (TAK 715, MAP kinase inhibitor I) and type II (BIRB796), as both types involve the ATP-binding site directly, but also of type III inhibitors (pyrazolourea) that causes conformational changes in the ATP-binding site upon binding to the allosteric site.

### 3.2. RAD Assays

Estrogen receptors are not only prime targets for hormone replacement therapies and subsequent anti-cancer investigations [11], but they are also targeted as a means of detecting endocrine disruptors [75]. Phytoestrogens are touted as possible alternatives to hormone replacement therapies without the side effects. To this end, Oosterkamp *et al.* [53] developed a RAD assay with recombinant human estrogen receptor steroid binding domain coupled to HPLC. Coumestrol was used as ligand, resulting in a major increase in fluorescence when it binds to the estrogen receptor (ER). Phytoestrogens could be detected in homogeneous or heterogeneous mode, where unbound coumestrol is retained on a restricted access column. In the chromatography-linked assay, the homogeneous configuration was used to prevent regeneration of the restricted access column by the organic modifier in the HPLC effluent. A dual knitted PTFE coil system was employed where the HPLC effluent was allowed to interact with the ER in the first coil and unbound ER to bind to coumestrol in the second coil at room temperature (Figure 2b). A mixture of seven human steroids (17-β-estradiol, progesterone, estrone, methyltestosterone, estriol, zeranol, diethylstilbestrol) was used as validation system for assay specificity and calculation of detection limits. Progesterone and 17-β-estradiol demonstrated the selectivity of the assay with progesterone not bound by the ER. The hydroxyl group of 17-β-estradiol that forms the recognition site for the ER is replaced with a carbonyl group in progesterone. The ER also did not bind methyltestosterone, as expected. Detecting 17-β-estradiol in spiked urine samples showed the applicability of this technique for future drug testing of athletes. This assay was also used as an illustration in two reviews on the theoretical aspects of ligand based HPLC on-line BCD assays [24] and xenoestrogens and endocrine disruptors [75], respectively.

Schobel *et al.* [11] developed the assay further to distinguish between ERα and ERβ. Coumestrol was still used as fluorescent ligand and the assay was set up in a homogeneous conformation. FIA mode and batch assays were used to screen for most active extracts from their library of plant extracts. Only the most active extracts were tested on the BCD system and ESI-MS was used to identify estrogenic compounds for which IC_50_ values were calculated. Using the IC_50_ values the binding affinities relative to 17-β-estradiol were calculated for identified compounds. Van Elswijk *et al.* [76] analyzed acid hydrolyzed pomegranate peel extracts for ERβ agonists and showed that the flavonoids, luteolin, quercetin and kaempferol, had agonistic properties. Due to an 80 s assay time with subsequent band broadening, highly concentrated extracts were analyzed and ELISA blocking reagent was used to prevent non-specific binding. Van Liempd *et al.* [13,30] combined metabolism assays and ER screening which is described in more detail in section 3.3. Kool *et al.* [54] used an analogous method to that of Oosterkamp *et al.* [53] to investigate pig and rat liver microsomal metabolites of tamoxifen and (*Z*)-4-hydroxytamoxifen for ERα affinity. Most of the 14 metabolites displayed various degrees of affinity for ERα. Reinen *et al.* [55] developed an ERα assay based on FP detection allowing fluorescence measurement at higher wavelengths. Fluorescence detection at these higher wavelengths negated the interference of auto-fluorescence of investigated compounds. A specific fluorescein-labeled estradiol derivative was synthesized as probe and the system optimized for detection purposes using a mixture of five known estrogenic compounds in FIA mode. De Vlieger *et al.* [8] performed a parallel assay for both human ERs on the metabolites of six estrogenic compounds, natural estrogen (17-α-estradiol and 17-β-estradiol), synthetic ER modulators, prodrugs (17-α-ethinylestradiol and norethisterone), and synthetic compounds from the Schlering-Plough library (ORG-X, ORG-Y). Estrogenic compounds were subjected to metabolism by human liver microsomes and mutant bacterial CYPs. Out of 85 metabolites, 55 hits on one or both ERs were detected and consisted of mostly hydroxylated or dihydroxylated and some dehydrogenated metabolites. ESI-MS was used for tentative structure elucidation, while preparative scale isolation of norethisterone metabolites enabled further structure elucidation by NMR. From these structures they could elucidate patterns of preferable metabolism sites on these compounds.

The urokinase plasminogen activator receptor (uPAR) is a multifunctional protein playing key roles in cellular adhesion and migration and is believed to regulate cellular responses during angiogenesis, inflammation, wound repair, and tumor metastasis [77]. Oosterkamp *et al.* [12] developed a RAD system for uPAR by synthesizing a solubilized fluorescent labeled uPAR. The proteolytic enzyme, urokinase plasminogen activator (uPA), was employed as a model protein to show the applicability of this RAD technique. Optimization was performed using FIA mode. No make-up flow was required and Tween 20 was used as additive in an assay performed at room temperature with a reaction time of 60 s. A specially designed affinity column was applied downstream of the reaction coil in heterogeneous mode to retain unbound receptor, as the receptor fluoresced in both bound and unbound states (Figure 2c). Hence, peaks on the fluorescence chromatogram were indicative of ligands bound to the uPAR. Plasma samples were analyzed for ligands of uPAR and active metabolites, but other complex matrices could in all probability be screened with this assay in search of therapeutic compounds of natural origin. This assay was set up as a model for similar types of assays to provide alternative methods to immunochemical assays which are less specific.

### 3.3. Application of EAD and RAD Assays in Metabolic Profiling Assays

Metabolite profiling systems are set up in a pre-chromatography configuration often including on-line SPE connected to HPLC (Figure 2d). Van Liempd *et al.* [29] developed a CYP 1A1/1A2 bio-reactor system with β-NF activated liver microsomes and ethoxyresorufin as fluorogenic substrate. The system, optimized for reaction temperature (37 °C), choice of SPE and substrates, included a PEEK filter to prevent blockages of the chromatographic system. Validation of the assay was performed by repeated analysis of the reaction products using a fluorescence detector. Regeneration of the SPE column and the filter device was performed with relative ease, which showed this to be a potentially valuable application for metabolite analysis.

The power of this assay was emphasized by two subsequent publications by the same authors, where the CYP bioreactor system was coupled to an ERα BCD assay to detect bioactivity of the metabolites. Two known selective estrogen receptor modulators, tamoxifen and raloxifene, metabolized by the CYP bioreactor system and analyzed for ERα affinity led to the identification of 15 active tamoxifen metabolites and six active raloxifene metabolites [13]. MS/MS analysis of the metabolites yielded more information, enabling identification of three novel raloxifene metabolites with activity. The CYP bioreactor system was also optimized for metabolites of endocrine disrupting compounds (EDCs) with an emphasis on xenoestrogens and their affinity for ERα [30]. Methoxychlor was used as candidate EDC with its metabolites, mono- and bis-hydroxymethoxychlor. Following successful optimization, the metabolism of 2-hydroxy-4-methoxy-benzophenone was investigated, resulting in three active metabolites with at least one novel hydroxyl metabolite. Even though this system was set up as a RAD assay for the detection of bioactive metabolites, it could also be used in conjunction with EAD or on-line antioxidant assays.

Recently, a biochip method using precision cut liver slices and a micro-fluidic technique was developed as alternative to microsomal assays [31]. The biochip system was not only stable under perfused conditions over a 24 h period, but it has several advantages, mainly because it contains whole cells and fully intact enzyme systems. The biochip system was tested with two substrates, 7-hydroxycoumarin and diclophenac, and one inhibitor, phloxine B, for which IC_50_ values could be calculated and thermo-unstable metabolites detected. In future this system might be coupled to an RAD, EAD or on-line antioxidant system to detect the bioactivity of drug metabolites resulting from liver biochips.

## 4. HPLC Coupled to On-Line Antioxidant Detection

HPLC on-line antioxidant assays have seen increasing application during the past few years for the investigation of plant extract constituents responsible for antioxidant activity. These assays can be classified, based on the principle of the assay, *i.e.*: (1) assays based on reaction with a stable oxidizing reagent; (2) assays based on reaction with a physiologically-relevant reactive oxygen species (ROS); and (3) assays based on electrochemical measurements. In the first type of assay, reaction of a stable oxidizing reagent with the antioxidant results in a measurable change (e.g., absorbance decrease or increase at a specific wavelength). The second type of assay uses an oxidizing agent which is also present in biological systems, *i.e.*, ROS such as superoxide radical anions or hydrogen peroxide. The ROS is able to oxidize a substrate, of which the concentration can be measured (e.g., using chemiluminescence). When an antioxidant counteracts the oxidation of the substrate, the change in concentration of unoxidized substrate can be measured. The third type of assay is based on the direct measurement of electron transfer from the antioxidant to an electrode, with both the measured potential and current providing information. HPLC on-line antioxidant assays were recently reviewed in detail by Niederländer *et al.* [78]. This section will aim to summarize the main findings of Niederländer *et al.* [78] with regard to the first two types of on-line antioxidant assays and will include methodological improvements and applications since 2008. On-line antioxidant assays based on electrochemical detection will not be covered in this review, as the focus is on post-column reaction with reagents aimed at detecting bioactivity of separated compounds.

### 4.1. On-Line Antioxidant Assays Based on Stable Oxidizing Reagents

On-line antioxidant assays using stable oxidizing reagents are generally simple and easy to handle compared to assays involving physiologically-relevant ROS. The basic instrumental configuration is the same for all antioxidant assays (Figure 2a). The only variation between assays is in the number of reagents added to the HPLC effluent. The most widely used assays of this type involve the stable free radicals, 1,1-diphenyl-2-picrylhydrazyl radical (DPPH^•^) and 2,2'-azinobis-(3-ethylbenzothiazoline-6-sulfonate) radical cation (ABTS^•+^), which are scavenged by antioxidants, resulting in a decrease in absorbance observed as negative peaks. Recently, an on-line radical scavenging assay, employing galvinoxyl radicals, has also been described [79]. Additionally, assays based on the redox reaction of antioxidants with a phosphomolybdate reagent [80], Folin-Ciocalteau reagent [81,82] and Cu(II)-neocuproine reagent [83], used in the cupric reducing antioxidant capacity (CUPRAC) batch assay, have been reported. An HPLC-chemiluminescence (CL) assay based on CL of acidic permanganate has also been reported [84–86], indicating reducing capacity of the antioxidants detected. In the latter four assays, the antioxidant compounds are detected as positive peaks due to formation of a colored product after reaction of the antioxidant with the reagent. Table 2 provides a comparison of several important aspects for each type of assay.

The first HPLC-DPPH assay, using a methanolic solution of DPPH^•^, was described by Koleva *et al.* [5]. The antioxidant compounds were detected as negative peaks at 517 nm. The DPPH^•^ concentration was optimized and the effect of reaction time, mobile phase composition and pH investigated. A reaction time of 30 s was chosen as optimum and the assay deemed applicable for gradient elution with a mobile phase composition varying between 10 and 90% organic modifier (methanol or acetonitrile) and pH 3 to 6.

Mobile phases at lower pH caused a dramatic loss of sensitivity due to a decrease in absorbance of DPPH^•^. Reaction temperature was not controlled. The reaction kinetics of compounds was considered to play a role as exemplified by differences in minimum detectable amounts for antioxidants known to be fast (quercetin; 6.6 ng) or slow (eugenol; 1.9 μg) DPPH^•^ scavengers. Dapkevicius *et al.* [87] improved the sensitivity up to 30-fold by adding citric acid-phosphate buffer at pH 7.6 to the methanolic reagent solution combined with degassing of the reagent solution, addition of a pulse damper and an increased reagent flow rate. Buffering of the reagent solution decreased the effect of acidic mobile phases. The reaction mechanism of the HPLC-DPPH assay was revisited by Bartasiute *et al.* [91], who showed that the presence of water in the mobile phase has a significant effect on the reaction kinetics compared to a pure methanol medium, mostly used in batch assays. The reaction of DPPH^•^ with an antioxidant is characterized by slow kinetics going to completion in methanol, while a dynamic equilibrium is established rapidly in the presence of water. This explains why several HPLC-DPPH applications use reaction times of less than 10 s compared to reaction times of 30 min to 2 h generally used in batch assays [84,91,92]. Bartasiute *et al.* [91] was also the first group to adopt a quantitative approach by calculating Trolox equivalent antioxidant capacity (TEAC) values for the antioxidant compounds, while thermodynamic equilibrium constants were also determined. Calculation of TEAC values were also reported by Arthur *et al.* [93], while De Beer *et al.* [4] calculated the antioxidant activity of hydroxycinnamic acid derivatives relative to that of caffeic acid. Most applications follow the protocol of either Koleva *et al.* [5] or Dapkevicius *et al.* [87] with minor modifications in terms of dimensions of the reaction coil, reagent flow rate and method of reagent solution delivery (syringe pump or HPLC pump) (as reviewed by Niederländer *et al.* [78]). A number of applications with modified methodology or novel approaches will be highlighted in the following text.

Application of the HPLC-DPPH assay using different buffers, such as citric acid-sodium citrate at pH 7.6 [94] or MES buffer at pH 6 [95], and higher temperatures, *i.e.*, 60 °C [84,95], has been reported. The HPLC-DPPH assay has been hyphenated with MS [79,96–102] and SPE-NMR [103,104] analysis by splitting the HPLC effluent before the on-line antioxidant reaction coil. He *et al.* [88] also applied the assay using normal phase HPLC (NP-HPLC) to identify non-polar tocopherols with antioxidant activity in a supercritical fluid extract of *Gardenia* fruit oil. Zhang *et al.* [79], on the other hand, investigated the use of NP-HPLC with on-line DPPH^•^ scavenging detection, but decided to rather use the galvinoxyl radical due to better signal-to-noise ratios. In both these cases, the reagent was prepared in hexane. Other novel applications include optimization of the assay for use with commercially available derivatization equipment [81,82] and use of the technique to obtain information on the antioxidant activity and the position of conjugation for hepatic phase II metabolites of aspalathin [105].

The first HPLC-ABTS assay was also developed by Koleva *et al.* [89], where antioxidant compounds were detected as negative peaks at 734 nm after reaction with the ABTS^•+^ reagent in a reaction coil giving a reaction time of 30 s. The reagent was prepared in 10% methanol in phosphate buffered saline (pH 7.4). The method was suitable for use with isocratic or gradient HPLC from 0 to 100% organic modifier at pH 3 to 7.4. The method was also optimized in terms of reagent concentration and reagent flow rate. The method proved to be more sensitive and more suitable for detection of water-soluble antioxidants than their HPLC-DPPH assay developed earlier [5]. Cano *et al.* [106] applied the method with minor modifications, namely preparation of the reagent using horse radish peroxidase and hydrogen peroxide, different coil dimensions and detection at 600 nm, while stressing the importance of a reaction time of at least 1 min to achieve complete reaction of all antioxidants. Detection is possible at various wavelengths due to multiple peaks in the spectrum of ABTS^•+^ [107]. Cano *et al.* [106] determined TEAC values for a range of antioxidants from hydrophilic (e.g., ascorbic acid) to lipophilic (e.g., tocopherols) antioxidants. TEAC values were also calculated by Stewart *et al.* [108] and He *et al.* [109], while De Beer *et al.* [4] calculated antioxidant values equivalent to caffeic acid for several hydroxycinnamic acid derivatives (HPLC-BCD chromatogram shown in Figure 1). Hyphenation of HPLC-ABTS with MS [110] and SPE-NMR [104,111] detection has also been reported. Novel methodology or modifications applied since 2008 include the following: NP-HPLC using ABTS^•+^ reagent prepared in phosphate buffered saline and diluted with methanol [88]; reaction temperature of 40 °C [108,112,113]; method validation [114]; assessment of the activity and position of conjugation for hepatic phase II metabolites of aspalathin [105]; and use of a commercially available derivatization equipment [81,82].

The only report of an on-line antioxidant assay employing galvinoxyl radicals is that of Zhang *et al.* [79] using NP-HPLC hyphenated with MS detection. The assay parameters, *i.e.*, reagent concentration, reagent solvent composition, flow rate and coil length, were optimized. A Superloop^®^ was employed for delivery of the reagent in hexane, which markedly increased the signal-to-noise ratio compared to reagent delivery using an HPLC pump. The method was applied to detect antioxidant tocopherols in wheat germ oil and olive oil, while carnosic acid, carnosol and an as yet unidentified compound were shown to be the major non-polar antioxidants in an oil-soluble rosemary extract.

Cardeñosa *et al.* [80] were the first to report an HPLC on-line antioxidant assay involving a phosphomolybdate reagent. No other reports mentioning this technique have been forthcoming until recent reports of an on-line antioxidant assay involving the related Folin-Ciocalteau reagent containing heteropolyphosphotungstates-molybdates [81,82]. Both these assays are believed to share a similar reaction mechanism involving electron-transfer from an antioxidant compound reducing Mo^6+^ to Mo^5+^ to form a blue-green complex detected at wavelengths higher than 580 nm [80,115]. High temperatures, *i.e.*, 95 °C and 130 °C, for the phosphomolybdate and Folin-Ciocalteau reagents, respectively, shortened the reaction time needed. The main technical difference between the assays involves the use of the commercially available Folin-Ciocalteau reagent and derivatization instrument by Kusznierewicz *et al.* [81,82] compared to the preparation of the reagent and the built-to-purpose reactor used by Cardeñosa *et al.* [80].

Cardeñosa *et al.* [80] optimized several parameters of their HPLC-phosphomolybdate assay, *i.e.*, HPLC flow rate, reagent flow rate, sodium phosphate concentration, ammonium molybdate concentration, sulfuric acid concentration and reaction temperature. The method was then applied to detect antioxidants, as well as to quantify tocopherol. Several tocopherols and carotenoids were identified as antioxidants in hexane extracts from lettuce, tomato, red pepper and soybean seed. The validated method for the quantification of tocopherols was shown to be more sensitive than a HPLC method with fluorescence detection. The reaction of antioxidants with the phosphomolybdate reagent is compatible with buffers and solvents normally used as HPLC mobile phases [116]. Interestingly, a reduction of reaction temperature to 27 °C in the on-line assay [80] or 37 °C in the batch assay [116] permitted some selectivity based on the nature of the antioxidant. At lower temperatures, only strong antioxidants showed a response.

The HPLC-Folin-Ciocalteau assay was also optimized in terms of reagent concentration and reaction temperature [81,82]. At the very high temperatures used in the assay (130 °C), some concern existed about the stability of antioxidant compounds during the assay (reaction time < 1 min), but no degradation was detected. Precipitation of salts in the Folin-Ciocalteau reagent was a problem when the reagent concentration was higher than 50% or the HPLC mobile phase contained more than 80% methanol. The presence of formic acid did not affect the stability of the reagent, although it affected the antioxidant activity of resveratrol compared to the batch assay. The optimized on-line assay was subsequently employed to determine TEAC values for mixtures of authentic standards including ascorbic acid and several phenolic compounds. The values were lower than those of the batch assay, but a strong correlation (>0.85) was observed between values obtained with the on-line and batch assays. Several antioxidant compounds were also identified in chokeberry, sloe and Mirabelle plum extracts.

The HPLC-CUPRAC assay, developed by Çelik *et al.* [83], makes use of electron-transfer from an antioxidant to the Cu(II)-neocuproine reagent resulting in its reduction to Cu(I)-neocuproine, which has an absorption maximum at 450 nm. The reaction of antioxidants with the CUPRAC reagent is reported to be unaffected by changes in solvent composition [117]. Apak *et al.* [118] also stated that the reaction is “relatively insensitive to a number of parameters, for example, air, sunlight, humidity, and pH to a certain extent”, although no data were shown to this effect. The CUPRAC reagent reacts rapidly with some compounds (< 20 min for complete reaction with amongst others ascorbic acid, gallic acid, ferulic acid and quercetin), but reactions with other compounds were slow (>20 min for complete reaction with naringin, naringenin and EGCG) [83,118,119]. Antioxidants with slow reaction kinetics may not be detectable using the on-line CUPRAC assay due to the short reaction time (<1 min). The on-line CUPRAC assay was successfully applied for the detection of antioxidants in *Camellia sinensis* (gallic acid and several flavan-3-ols), *Origanum marjorama* (hydroxycinnamic acids, flavonols and flavone aglycones) and *Mentha* (rosmarinic acid and flavone aglycones) extracts [83]. Very little band-broadening was observed, presumably due to the short reaction time using a coil with narrow-bore tubing. Very low limit of detection values were obtained due to a low noise level. Some baseline drift was observed in the area of the chromatogram corresponding to a high concentration of organic solvent, but this could be taken into account by subtracting a blank chromatogram.

HPLC-CL detection of antioxidants based on an acidic potassium permanganate system has been described recently [84–86,90]. The exact mechanism for redox reaction between permanganate and antioxidant compounds at low pH is not yet clear [120], but the reaction results in light emission due to an electronically excited Mn(II) species. Antioxidants are therefore detected as positive peaks in the HPLC-CL chromatogram based on their reducing capacity. Sodium polyphosphate or sodium hexametaphosphate is added as CL enhancer in the reported assays. The reaction is extremely rapid needing no reaction coil, which is a prerequisite for other on-line antioxidant assays. The CL is detected immediately after mixing of the HPLC effluent with the acidic potassium permanganate reagent using a flow-through luminometer. The experimental set-up is, therefore, much simpler and cheaper than other on-line antioxidant assays. Parameters such as reagent and enhancer concentration, pH and flow rate were optimized to obtain the best CL response [86]. The only drawback of the method is that the CL signal is significantly quenched by acetonitrile, dictating the use of methanol gradients. Acidification of the aqueous HPLC mobile phase did not have a significant effect on the signal intensity. Interesting applications using this method include parallel detection of DPPH^•^ scavenging activity and CL after splitting the HPLC effluent [84], as well as two dimensional HPLC hyphenated to CL detection of antioxidant compounds [85].

### 4.2. On-Line Antioxidant Assays Based on Physiologically-Relevant ROS

**Figure 3:**
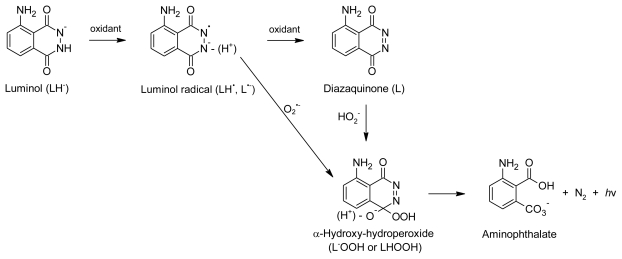
Reaction mechanism for the oxidation of luminol (5-amino-2,3-dihydrophthalazine-1,4-dione) to produce chemiluminescence (adapted from Rose & Waite [123]).

Most assays based on physiologically relevant ROS rely on CL at 425 nm produced by oxidation of luminol (5-amino-2,3-dihydrophthalazine-1,4-dione) to 3-aminophthalate (Figure 3) [121]. Luminol can be oxidized by hydrogen peroxide and/or superoxide radical anion depending on the ROS generation system used [78]. A catalyst, such as various enzymes, metalloproteins, metal ions or metal ion complexes, is needed for the reaction [122]. Antioxidant compounds cause a decrease in CL due to scavenging of the ROS or interference with the oxidation of luminol, although in some cases inhibition of an enzyme, which produces the ROS or acts as a catalyst, can also play a role [78].

As most mechanisms involved in CL quenching in the HPLC-CL assays are related to an aspect of antioxidant activity, activity in these assays should be seen as an indication of the general electron donating ability of an antioxidant. Many HPLC-CL assays based on luminol oxidation have been reported for the detection of compounds without reference to their antioxidant activity as reviewed by Gámiz-Gracia *et al.* [124]. The present review, however, will focus solely on applications specifically developed for detection of antioxidant compounds. Applications involving ROS, but not chemiluminescence, have also been reported. A rather complex system detecting both antioxidants and pro-oxidants (termed pro-oxidant and antioxidant detection, PAD) was reported by Kool *et al.* [9]. On-line antioxidant assays based on physiologically-relevant ROS are summarized in Table 3 in terms of ROS generation system, detection and probable mechanisms involved, as well as suitability of HPLC mobile phases. HPLC-CL assays generally follow the instrumental configuration depicted in Figure 2a, but with the addition of a second pump delivering a second reagent before the reaction coil for most of the published assays.

The first HPLC-CL method developed specifically for detection of antioxidant compounds by Dapkevicius *et al.* [125] used hydrogen peroxide as oxidant and MP11 as catalyst for luminol oxidation at pH 10. The method was successfully applied to identify antioxidant compounds in sage and thyme extracts using gradient elution. A similar method using only luminol and hydrogen peroxide at pH 8 was reported by Toyo’oka *et al.* [126]. Ding *et al.* [127] used a system similar to that of Dapkevicius *et al.* [125], but ethylenediaminetetraacetic acid was included in the reaction mixture, presumably to prevent interference by iron. These assays purport to detect antioxidant compounds mainly on the basis of hydrogen peroxide scavenging activity, although other mechanisms may play a role as highlighted above.

On-line detection of compounds with superoxide radical anion scavenging activity has been performed using a hypoxanthine/xanthine oxidase system in the presence of catalase to prevent participation of hydrogen peroxide in the reaction [126,128].

Both assays used luminol to produce CL after oxidation by the superoxide radical anion. Ogawa *et al.* [128] included potassium hexacyanoferrate as catalyst, added as a third reagent before the reaction coil. In these two methods, inhibition of xanthine oxidase by detected compounds cannot be excluded. Superoxide radical anion generation using autoxidation of pyrogallol in alkaline medium (pH 11) has also been used in a HPLC-CL assay involving luminol [127].

The HPLC-PAD assay described by Kool *et al.* [9] involves generation of both superoxide radical anion and hydrogen peroxide, detected using a fluorescence reaction. The system is quite complex, but can detect both antioxidants and pro-oxidants. In a process catalyzed by cytochrome P450s and cytochrome P450 reductase, pro-oxidants in the HPLC effluent are converted by NADPH to radicals that react with oxygen to form superoxide anion radicals. In turn, the superoxide radical anion is converted by superoxide dismutase to hydrogen peroxide, which together with horse radish peroxidase reacts with 4-hydroxyphenylacetic acid to form a fluorescent product. In order to detect antioxidants, a pro-oxidant like paraquat is included in the reagent to give a baseline fluorescence that can be quenched by antioxidants in the HPLC effluent. In this scenario, antioxidants can react both with the superoxide radical anion generated or the reduced pro-oxidant, while direct reaction with hydrogen peroxide and inhibition of most of the enzymes involved are also possible.

## 5. Conclusions

Development of on-line HPLC-BCD assays provides researchers working in the field of pharmacognosy with a number of methodologies to fast-track identification of interesting and/or novel bioactives. In future it could become a strategic tool in the arsenal of pharmacognosists. Combined with MS detection and even NMR, the long and tedious process of bioassay-guided fractionation and isolation for structure elucidation could be shortened even further. In spite of the advantages, on-line HPLC-BCD assays presently remain the domain of a limited number of research groups as “plug-and-play” systems are not yet commercially available and the number of methodologies available in on-line format are still limited. The use of commercial derivatization equipment in some on-line antioxidant assays makes the application of these assays more accessible.

## Figures and Tables

**Table 1:** Summary of HPLC-EAD and -RAD assays with emphasis on detection methods and relevant therapeutic areas of bioactives.

Assay	Detection	Relevance
*EAD assays*		

Acetylcholinesterase inhibitors [6,7,38–41]	UV-Vis, 405 nm [6,7,38] FL, λ_ex_ 406nm, λ_em_ 505 nm [39,40] ESI-MS [41]	Treatment of Alzheimer’s disease, senile dementia, Parkinson’s disease, ataxia and myasthenia gravis
α-Glucosidase inhibitors [14,42]	UV-Vis, 405 nm [14,42]	Treatment of diabetes type II
Angiotensin 1 converting enzyme [10]	FL, λ_ex_ 320 nm, λ_em_ 420 nm [10]	Treatment of hypertension, cardiac disease, diabetic nephropathy
Liver cytochrome P450 ligands [15,43–45]	FL, λ_ex_ 530 nm, λ_em_ 586 nm [15,43] FL, λ_ex_ 530 nm, λ_em_ 590 nm [44] FL, λ_ex_ 530 nm, λ_em_ 580 nm [45]	Cancer prevention
Glutathione-*S*-transferase inhibitors [16,46]	FL, λ_ex_ 290 nm, λ_em_ 465 nm [16,46]	Enhancement of anticancer treatments
Serine protease inhibitors [47]	FL, λ_ex_ 342 nm, λ_em_ 440 nm [47]	Treatment of thrombosis
HIV-protease inhibitor [48,49]	FL, λ_ex_ 340 nm, λ_em_ 490 nm [48,49]	Treatment of AIDS
Phosphodiesterase inhibitors [50]	FL, λ_ex_ 280 nm, λ_em_ 460 nm [50]	Treatment of hypertension, vascular conditions and asthma
Kinase/phosphatase inhibitors [51]	FL, λ_ex_ 425 nm, λ_em_ 464 nm [51]	Novel drug targets
MAP-kinase inhibitors [52]	FL, λ_ex_ 355 ± 4 nm, λ_em_ 405 ± 5 nm [52]	Treatment of inflammatory diseases

*RAD assays*		

Estrogen receptor ligands [8,11,13,30,53–55]	FL, λ_ex_ 340 nm, λ_em_ 410 nm [8,11,13,30,53,54] FP, λ_ex_ 485 nm, λ_em_ 520 nm [55]	Hormone replacement therapy, chemoprevention, detecting endocrine disruptors
Urokinase plasminogen activator receptor ligands [12]	FL, λ_ex_ 489 nm, λ_em_ 520 nm [12]	Important role in angiogenesis, inflammation, wound repair and tumor metastasis

AIDS, acquired immune deficiency syndrome; ESI-MS, electrospray ionization mass spectrometry; FL, fluorescence; FP, fluorescence polarization; HIV, human immunodeficiency virus; UV-Vis, ultraviolet and visual spectrum.

**Table 2:** Comparison of on-line antioxidant assays based on reaction with a stable oxidizing reagent.

Assay	Reaction Mechanism	Detection	Reagent Solution Characteristics	HPLC Mobile Phase Compatibility
DPPH^•^ scavenging [5,79,87,88]	H-donation	UV-VIS, 510–520 nm	DPPH^•^ in MeOH or MeOH/buffer (pH 7.6) mixture for RP-HPLC; *n*-hexane for NP-HPLC	10–90% organic modifier at pH 3–6 a for RP-HPLC; gradient of *n*-hexane and isopropanol for NP-HPLC
ABTS^•+^ scavenging [88,89]	e^−^-transfer	UV-VIS, 410–430, 630–640, 734 nm	ABTS^•+^ in buffer or MeOH/buffer mixture (pH 7.4or 7.6) for RP-HPLC; MeOH for NP-HPLC	0–100% organic modifier at pH 3–7.4 (TFA not recommended) for RP-HPLC; gradient of *n*-hexane and isopropanol for NP-HPLC
Galvinoxyl^•^ scavenging [79]	H-donation	UV-VIS, 425 nm	Galvinoxyl^•^ in 100% *n*-hexane or MTBE	Suitable for NP-HPLC using gradient of *n*-hexane and MTBE
Phosphomolybdate/Folin-Ciocalteau reagent [80–82]	e^−^-transfer	UV-VIS, 598, 750 nm	Phosphomolybdate/Folin-Ciocalteau reagent in acidic aqueous solution	Suitable for use with most RP-HPLC solvents; <80% organic modifier to prevent precipitation of salts; not suitable for NP-HPLC as reagent not soluble in 100% organic mobile phase
CUPRAC reagent [83]	e^−^-transfer	UV-VIS, 450 nm	Cu(II)-neocuproine in ammonium acetate buffer (pH 7)	Suitable for use with most RP-HPLC solvents; not suitable for NP-HPLC as reagent not soluble in 100% organic mobile phase
Acidic KMnO_4_ reagent [84–86,90]	unknown	CL	KMnO_4_ and Na polyphosphate or Na hexametaphosphate (enhancer) solution, adjusted to pH 2 or 2.3 with H_2_SO_4_	Acidified aqueous phases combined with MeOH gradients; MeCN not recommended due to CL quenching

a lower pH mobile phases can be used when buffering the reagent solution;

ABTS, 2,2'-azinobis-(3-ethylbenzothiazoline-6-sulfonate); CL, chemiluminescence; CUPRAC, cupric reducing antioxidant capacity; DPPH, 1,1-diphenyl-2-picrylhydrazyl; MeCN, acetonitrile; MeOH, methanol; MTBE, methyl *tert*-butyl ether; NP-HPLC, normal phase high performance liquid chromatography; RP-HPLC, reversed phase high performance liquid chromatography; TFA, trifluoroacetic acid.

**Table 3:** Summary of on-line antioxidant assays based on physiologically relevant reactive oxygen species (ROS).

Assay	Reaction Mechanism	Detection	Reagent Solution Characteristics	HPLC Mobile Phase Compatibility
*Assays based on H_2_O_2_scavenging*				

HPLC-CL H_2_O_2_/MP11/ luminol [125]	Oxidant: H_2_O_2_; Catalyst: MP11; Emitter: luminol oxidation product	CL, 425 nm	Reagent 1: MP11 and luminol in 30% methanol/buffer (pH 10); Reagent 2: aqueous H_2_O_2_	No addition of acidifier; acetonitrile content ≥ 30%
HPLC-CL H_2_O_2_/luminol [126]	Oxidant: H_2_O_2_; Emitter: luminol oxidation product	CL	Reagent 1: luminol in 10% methanol/buffer (pH 8); Reagent 2: aqueous H_2_O_2_	Isocratic elution with MeOH/1% H_3_PO_4_ (28/71); eluent neutralized before addition of CL reagents
HPLC-CL H_2_O_2_/EDTA/ luminol [127]	Oxidant: H_2_O_2_; Emitter: luminol oxidation product	CL	Reagent 1: luminol and EDTA in buffer (pH 10); Reagent 2: aqueous H_2_O_2_	0.1% H_3_PO_4_ aqueous phase combined with MeCN gradient < 65%; higher acid concentration or MeOH gradient caused baseline drift

*Assays based on O_2_^•−^scavenging*				

HPLC-CL HX/XOD/catalase/ K_3_Fe(CN)_6_/ luminol [128]	Oxidant: O_2_^•−^ (HX/XOD/catalase) Catalyst: K_3_Fe(CN)_6_; Emitter: luminol oxidation product	CL	Reagent 1: HX and luminol in 10% methanol/buffer (pH 8); Reagent 2: aqueous K_3_Fe(CN)_6_; Reagent 3: XOD and catalase in buffer (pH 8)	No addition of acidifier; MeOH/water gradient
HPLC-CL HX/XOD/catalase/ luminol [126]	Oxidant: O_2_^•−^ (HX/XOD/catalase) Emitter: luminol oxidation product	CL	Reagent 1: HX and luminol in 10% methanol/buffer (pH 8); Reagent 2: XOD and catalase in buffer (pH 8)	Isocratic elution with MeOH/1% H_3_PO_4_ (28/71); eluent neutralized before addition of CL reagents
HPLC-CL pyrogallol/EDTA/ luminol [127]	Oxidant: O_2_^•−^ (pyrogallol); Emitter: luminol oxidation product	CL	Reagent 1: luminol and EDTA in buffer (pH 11); Reagent 2: aqueous pyrogallol	0.1% H_3_PO_4_ aqueous phase combined with MeCN gradient < 65%; higher acid concentration or MeOH gradient caused baseline drift
HPLC-PAD [9]	Oxidant: O_2_^•−^ (CYPs/CYP reductase/HRP/ SOD/NADPH); FL-probe: 4-HPAA	FL, λ_ex_ 320 nm, λ_em_ 409 nm	Reagent 1: CYPs, CYP reductase, HRP and SOD in buffer (pH 7.8); Reagent 2: NADPH and 4-HPAA in buffer (pH 7.8)	Make-up flow with reverse gradient added

CYP, cytochrome P450; EDTA, ethylenediaminetetraacetic acid; FL, fluorescence; 4-HPAA, 4-hydroxyphenylacetic acid; HPLC-CL, high performance liquid chromatography-chemiluminescence; HRP, horseradish peroxidase; HX, hypoxanthine; MeCN, acetonitrile; MeOH, methanol; MP11, microperoxidase-11; NADPH, reduced β-nicotinamide adenine dinucleotide phosphate; PAD, pro-oxidant and antioxidant detection; SOD, superoxide dismutase; XOD, xanthine oxidase.
